# Marine-Derived Fucoidan Modulates Pathways Associated with Age-Related Macular Degeneration in Cellular and Zebrafish Models

**DOI:** 10.3390/md24060216

**Published:** 2026-06-16

**Authors:** Haqdil Hakeem Shad, Philipp Dörschmann, Samira Laura Hautmann, Johann Roider, Alexa Klettner

**Affiliations:** Department of Ophthalmology, University Medical Center, University of Kiel, Arnold-Heller-Str. 3, Haus 25, 24105 Kiel, Germany; philipp.doerschmann@uksh.de (P.D.); alexa.klettner@uksh.de (A.K.)

**Keywords:** fucoidan, polysaccharide, porcine retinal pigment epithelium (RPE), innate immunity, neurodegenerative diseases, age-related macular degeneration (AMD), zebrafish (*Danio rerio*), gene expression, vascular endothelial growth factor A (VEGF-A), nitric oxide (NO), reactive oxygen species (ROS)

## Abstract

Fucoidan, a sulfated polysaccharide, is known for its beneficial bioactive effects, for example antioxidant, anti-inflammatory, and vascular modulatory effects. Such a bioactive compound may also be useful for treating neurodegenerative diseases like age-related macular degeneration (AMD). Our research focuses on AMD-related pathomechanisms using primary porcine retinal pigment epithelium (RPE) cells in vitro and zebrafish (*Danio rerio*) models in vivo. We tested the bioactivity of a commercially available fucoidan (FVs) from bladderwrack with regard to pathomechanisms of AMD. We performed multiplex assays, RT-qPCR and fluorescence-based assays for the formation of nitric oxide (DAF-FM assay) and reactive oxygen species (DCF-DA assay) to analyze angiogenesis-related chemokines and pro-inflammatory cytokines as well as protection against oxidative stress and inflammatory insult. Our results showed that FVs significantly reduced the secretion of pro-angiogenic vascular endothelial growth factor A (VEGF-A) and follistatin as well as the pro-inflammatory cytokines interleukin 8 (IL-8) after lipopolysaccharide (LPS) and polyinosinic/polycytidylic acid (PIC) induction. Interleukin 6 (IL-6) was also reduced in the supernatant of the RPE cells. Additionally, in zebrafish, fucoidan decreased the production of NO and ROS. Gene expression of zebrafish embryos revealed anti-inflammatory effects by suppressing pro-inflammatory genes and significantly downregulating, e.g., interleukin 1 beta (IL-1β). These findings indicate modulation of oxidative stress, inflammatory responses, and VEGF secretion of the used FVs. This study demonstrates that fucoidan possesses AMD-relevant bioactivities in vitro and in vivo, suggesting fucoidan warrants further investigation in AMD-related research and related pathological mechanisms.

## 1. Introduction

Age-related macular degeneration (AMD) is an irreversible, multifactorial disease that commonly affects elderly individuals in industrialized nations and leads to severe vision loss. In 2020, approximately 196 million people were affected by AMD, which is estimated to be 8.69% of the world’s population. This number is expected to rise to 288 million by 2040 [1]. Due to its complex and multifactorial nature, the exact pathogenesis of AMD remains incompletely understood [2,3]. AMD is classified into an asymptomatic early stage and two vision-threatening late forms. The late dry form, known as atrophic AMD, is characterized by degeneration of the retinal pigment epithelium (RPE) and photoreceptors, resulting in gradual vision loss. The late stage, known as exudative AMD, involves pathological neovascularization within the retina, leading to rapid and severe vision impairment. Currently, no curative treatment exists for AMD. For wet AMD, intravitreal injections of vascular endothelial growth factor (VEGF) inhibitors and their derivatives are used, but these therapies only slow disease progression and do not stop it, and require high patient compliance [4].

The pathology of AMD primarily affects the RPE cells. This includes their function in the tissue and cellular levels; in addition, the pathomechanisms underlying AMD development mainly involve oxidative stress, inflammation, and angiogenesis. Among these, angiogenesis plays a central role in wet AMD, where abnormal blood vessel formation is initiated by excessive secretion of VEGF [4]. The RPE cells perform multiple essential functions to support photoreceptor survival and maintain visual function [5]. Under oxidative stress conditions in the retina, oxidation-specific compounds are formed that may trigger inflammatory responses. Such stress-induced inflammatory responses increase with age and greatly contribute to AMD progression [6].

**Figure 1:**
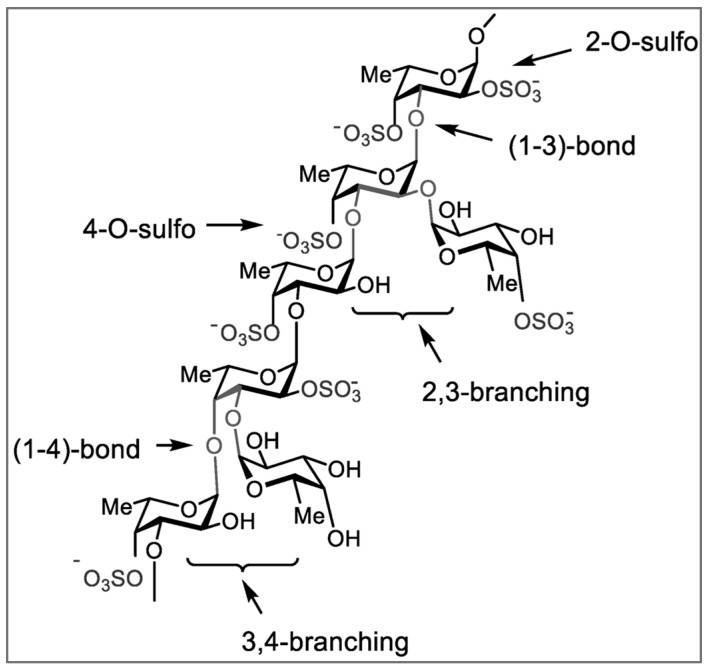
Schematic representation of a typical fucoidan structure featuring α-(1→3)- and α-(1→4)-linked L-fucose residues containing sulfate groups, as well as branching points. Structural features vary according to algal species, extraction methods, and environmental conditions. Figure modified from [10].

An active ingredient that targets the mechanisms causing AMD would be useful to stop the early progression of AMD development and to prevent severe forms. A potential therapeutic bioactive marine polysaccharide known as fucoidan has shown promising effects against several of the pathways involved in AMD pathogenesis. Fucoidans are structurally heterogeneous polysaccharides, and their biological activities strongly depend on the seaweed species and the molecular formation of the compound (Figure 1) which is influenced by the environmental factors [7,8,9,10]. The brown seaweed species *Fucus vesiculosus* is well studied and is known for high fucoidan content [11].

Various types of fucoidan have previously been tested in retinal cells and organ culture models for their antioxidative, anti-inflammatory and anti-angiogenic effects [12]. Given the considerable therapeutic potential of fucoidan for AMD, a comprehensive in vivo approach should be prioritized to expand current knowledge which could lead to potential therapeutic uses of fucoidan.

The zebrafish (*Danio rerio*) has a visual system that is highly comparable to that of other vertebrates, making it a valuable model for visual neuroscience research. The anatomical, and genetic components of visual processing have been well studied in both embryonic and adult stages [13,14]. The zebrafish retina shares strong similarities with the human retina in terms of cellular composition, phototransduction mechanisms, and metabolic function. More importantly, zebrafish embryos lack a fully developed adaptive immune system, which makes them particularly suitable for investigating innate immune-mediated ocular diseases and age-related degeneration. Such models have already been used for inherited retinal diseases and AMD experiments [14,15,16,17,18,19,20,21,22].

While previous studies have primarily investigated fucoidan in vitro retinal cell models, in vivo validation of AMD-relevant mechanisms remains limited. Therefore, combining cellular and organismal approaches may provide improved insight into pathway-level effects and enhance translational relevance. Several studies have demonstrated anti-inflammatory, antioxidant, and anti-angiogenic activities of fucoidans in retinal and retinal pigment epithelium cell models. However, most available evidence is derived from in vitro systems, and the in vivo validation of AMD-relevant pathway modulation by fucoidans remains limited. In particular, the effects of commercially available *Fucus vesiculosus* fucoidan on inflammatory, oxidative stress-related, and angiogenic pathways have not been comprehensively investigated in complementary in vivo models. Therefore, the present study combines primary porcine RPE cells with zebrafish models to evaluate AMD-relevant biological activities across both cellular and organismal systems.

In this study, we investigated the bioactivity of commercially available fucoidan derived from *F. vesiculosus* and evaluated its angiogenic, inflammatory, and oxidative stress-related effects using 1. in vitro experiments on freshly isolated and cultured primary porcine RPE cells and 2. in vivo experiments using transgenic- and wild-type zebrafish (*Danio rerio*) models relevant to AMD and associated pathways. This combined approach aims to bridge the gap between established in vitro RPE findings and limited in vivo evidence, thereby advancing the research of fucoidan as a potential therapeutic candidate for AMD and relevant diseases.

This study does not aim to model a specific AMD subtype, but rather to investigate key pathomechanisms including angiogenesis, inflammation, and oxidative stress. The zebrafish model is used as an in vivo screening system to evaluate these pathways rather than as a full model of retinal aging or chronic AMD pathology.

## 2. Results

### 2.1. VEGF and Angiogenesis-Related Biomarkers in Primary RPE Cells

We investigated the effect of fucoidan from *F. vesiculosus* (FV) on intracellular VEGF production and its secretion into the cell culture supernatant.

Primary porcine RPE cells were cultured and treated with FV (50 µg/mL) for 3 days. Subsequently, cell supernatant and lysates were collected for quantitative analysis of angiogenesis-related biomarkers, which are directly or indirectly involved in the angiogenesis processes including follistatin (FST), insulin-like growth factor binding protein 1 (IGFBP-1), VEGF-A, vascular endothelial growth factor receptor 1/Fms-like tyrosine kinase 1 (VEGF-R1 (FLT-1), and vascular endothelial growth factor receptor 2/Kinase insert domain receptor (VEGF-R2/KDR). Protein secretion levels were quantified and compared with untreated control groups using multiplex assays.

**Figure 2:**
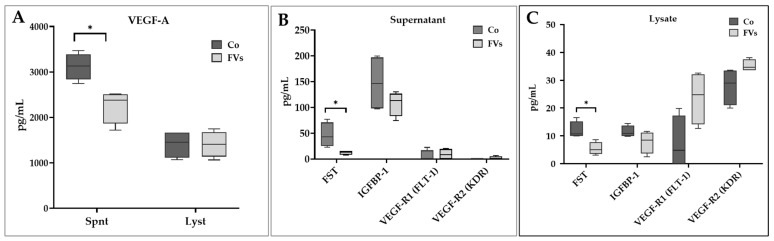
Effects on VEGF-related signaling of FV (fucoidan) in supernatant and lysate of the retinal pigment epithelial (RPE). (**A**) Vascular endothelial growth factor (VEGF-A) secretion in the culture supernatant (Spnt) and cell lysate (Lyst). Secretion of follistatin (FST), insulin-like growth factor binding protein 1 (IGFBP-1), vascular endothelial growth factor receptor 1/Fms-like tyrosine kinase 1 (VEGF-R1 (FLT-1)), vascular endothelial growth factor receptor 2/kinase insert domain receptor (VEGF-R2 (KDR)), (**B**) in the cell culture supernatant and (**C**) lysate of the RPE cells following fucoidan treatment and multiplex assay. For non-parametric data, Mann–Whitney test was applied for statistical analysis (*p*-value = * 0.05); Co (control); *n* = 5.

FV treatments resulted in a significant reduction in VEGF-A secretion in the supernatant, decreasing from 3119.64 ± 295.08 pg/mL (control) to 2251.36 ± 366.63 pg/mL (FV) with (*p*-value = 0.030, Figure 2A). No reduction in intracellular VEGF-A levels was observed in RPE cell lysates. Intracellular VEGF-A levels were unchanged, while extracellular VEGF-A levels were reduced, indicating a potential alteration in secretion or stability rather than expression. These findings suggest that FV may influence VEGF-A secretion, extracellular availability, or stability rather than directly suppressing VEGF-A production.

FVs also significantly reduced FST levels in both supernatant and lysates. The FST concentrations decreased from 46.57 ± 23.79 pg/mL (control) to 12.28 ± 3.73 (FV) pg/mL in the supernatant (*p*-value = 0.030) and from 11.97 ± 3.11 pg/mL (control) to 5.42 ± 2.31 pg/mL in the lysate of the cells (*p*-value = 0.030, Figure 2B,C).

A reduction in IGFBP-1 secretion was also observed in the supernatant as well as in the lysates of the cells following the FV treatment. This effect, however, did not reach statistical significance. No inhibitory effects of FV on VEGF-R1 (FLT-1) or VEGF-R2 (KDR) were detected in either supernatants (Figure 2B) or cell lysates (Figure 2C). A trend towards an increase was observed for VEGF-R1 (FLT-1) and VEGF-R2/KDR in the cell lysate, but this was not statistically significant. This increase, however, was not seen in the cell supernatant.

### 2.2. Inflammatory Cytokines in the RPE

#### 2.2.1. Inflammatory Cytokines in the Cell Supernatant

In this study, we examined the impact of FV on cytokine secretion under pro-inflammatory conditions. Primary RPE cells were cultured for at least 2 weeks prior to treatments. RPE cells were pretreated with FV (50 µg/mL) for 30 min and subsequently stimulated with lipopolysaccharide (LPS, 10 µg/mL), polyinosinic:polycytidylic acid (PIC, 10 µg/mL), or erastin (25 µM) for 3 days. The concentrations of these stimuli were selected based on previous studies [23]. For comparison, appropriate untreated, FV-only, stimulus-only and FV + stimulus were included. Cytokine secretion levels in the supernatant and cell lysates were quantified using ProcartaPlex assays. Measured cytokines included interferon alpha (IFNα), interferon gamma (IFNγ), tumor necrosis factor alpha (TNFα), interleukin-1 beta (IL-1 β), interleukin-4 (IL-4), interleukin-6 (IL-6), interleukin-8 (IL-8/CXCL8), interleukin-10 (IL-10), and interleukin-12 (IL-12/IL-23p40).

**Figure 3:**
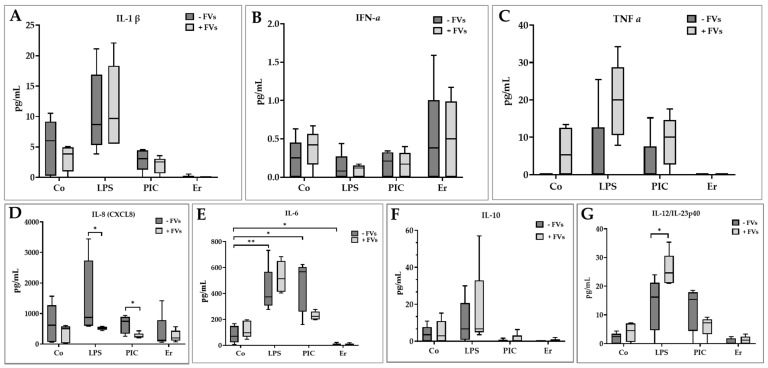
Inflammatory cytokine secretion levels in the culture supernatant of retinal pigment epithelial (RPE) after fucoidan (FV) treatment. Lipopolysaccharide (LPS) and polyinosinic/polycytidylic acid (PIC) and Erastin (Er) were used as stressors. The analyzed cytokines included (**A**) interleukin-1 beta (IL-1β), (**B**) interferon alpha (IFNα), (**C**) tumor necrosis factor alpha (TNFα), (**D**) interleukin-8 (IL-8/CXCL8), (**E**) interleukin-6 (IL-6), (**F**) interleukin-10 (IL-10), and (**G**) interleukin-12/23 p40 (IL-12/IL-23p40). For non-parametric data, statistical significance was determined using the Mann–Whitney test. Significance levels are indicated as *p*-value = * 0.05, ** 0.01, Co = control, *n* = 5.

The results show a selective, stimulus-dependent modulation in the secretion of IL-8 in response to LPS and PIC stimulation. The IL-8 secretion level was reduced from 1507.83 ± 1232.36 (LPS) pg/mL to 514.46 ± 52.32 pg/mL (FV) against LPS-stimulated cells (*p*-value = 0.020) and from 637.05 ± 290.83 pg/mL (PIC) to 268.83 ± 91.49 pg/mL (FV) against PIC-stimulated cells (*p*-value = 0.030). This corresponds to reductions of more than 65% and 57% of IL-8 respectively (Figure 3D). This suggests that FV primarily influences cytokine secretion rather than intracellular cytokine production.

The FV treatment also resulted in a reduction in IL-6 secretion against PIC-stimulated cells. The IL-6 secretion was reduced from 460.46 ± 196.50 pg/mL (PIC) to 229.07 ± 32.48 pg/mL (FV) corresponding to more than 50% inhibition; however, this did not reach statistical significance (Figure 3).

In contrast, FV significantly increased IL-12 secretion levels compared to LPS-stimulated cells from 13.57 ± 9.25 pg/mL (LPS) to 25.61 ± 5.84 pg/mL (FV) with *p*-value = 0.030. On the other hand, the FV reduced IL-12 secretion compared to PIC-stimulated cells; however, the reduction was not statistically significant. No major effects of FV were observed for IFNα, IFNγ, TNFα, IL-1β, IL-4 or IL-10 secretion (Figure 3). The IFNγ and IL-4 levels were below the detection limit in the supernatants and therefore not included in Figure 3. Overall, these findings indicate selective and stimulus-dependent immunomodulatory effects of FV.

#### 2.2.2. Cytokine Secretion Level in the Cell Lysates

**Figure 4:**
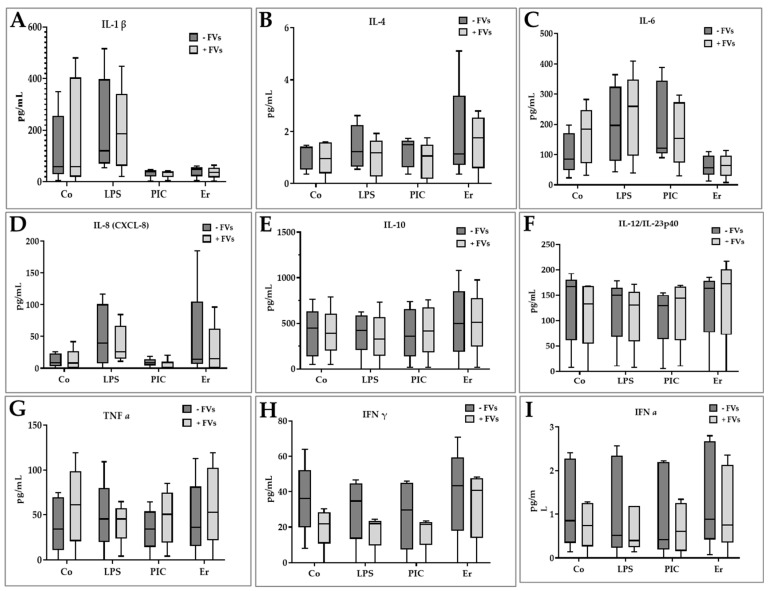
Inflammatory cytokine secretion levels in the lysate of retinal pigment epithelial (RPE) after fucoidan (FV) treatment. Lipopolysaccharide (LPS) and polyinosinic/polycytidylic acid (PIC) and erastin (Er) were used as pro-inflammatory agents. The analyzed cytokines included (**A**) interleukin-1 beta (IL-1β), (**B**) interleukin-4 (IL-4), (**C**) interleukin-6 (IL-6), (**D**) interleukin-8 (IL-8/CXCL8), (**E**) interleukin-10 (IL-10), (**F**) interleukin-12 (IL-12/IL-23p40), (**G**) interferon alpha (IFNα), (**H**) interferon gamma (IFNγ), (**I**) tumor necrosis factor alpha (TNFα). For non-parametric data, statistical significance was determined using the Mann–Whitney test, Co = control, *n* = 5.

FV treatment did not induce major changes in cytokine levels within RPE cell lysates. Nevertheless, a trend towards reduced IFNγ levels was observed in LPS and PIC-stimulated cells following treatment with FV. The IFNγ level was reduced from 30.22 ± 18.53 pg/mL (LPS) to 17.63 ± 10.04 pg/mL (FV) and from 26.93 ± 19.66 pg/mL (PIC) to 17.35 ± 9.79 pg/mL (FV). Similarly, IL-8 protein levels showed a non-significant reduction when treated with FV compared with LPS-stimulated cells. IL-8 was reduced from 51.25 ± 48.69 pg/mL (LPS) to 37.94 ± 29.71 pg/mL (FV). No other major differences in cytokine secretion was found in the lysate of the RPE cells (Figure 4).

### 2.3. In Vivo Gene Expression

To further investigate the potential anti-inflammatory effects of FV, we used zebrafish embryos for in vivo gene expression. Two-day-post-fertilization (2-dpf) embryos were treated with FV (50 µg/mL) and/or LPS (30 µg/mL) for 3 hours. These parameters were chosen after optimization. After 3 hours of treatment embryos were subjected to RNA isolation, reverse transcribed to cDNA, and gene expression was analyzed by real-time qPCR targeting 96 genes associated with inflammation, oxidative stress, and VEGF-related pathways including multiple endogenous controls. The broad gene expression screening approach was considered to be exploratory. Statistical analysis was performed using Student’s *t*-test provided from ThermoFisher connect.

**Figure 5:**
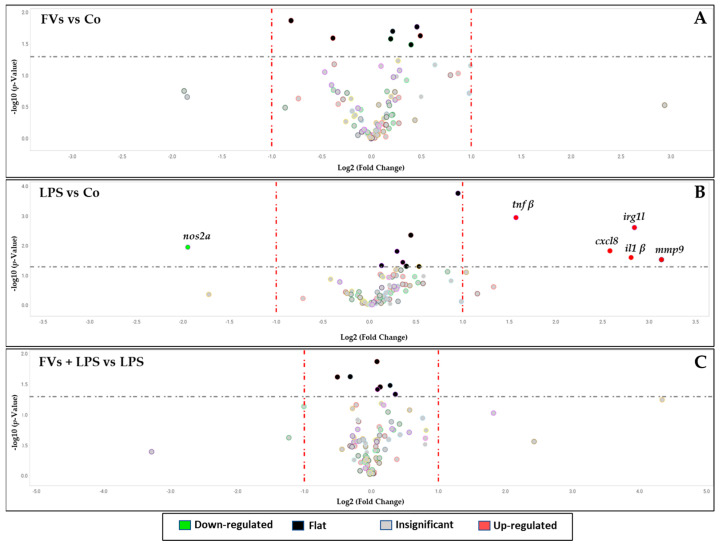
Volcano plot showing the effects of fucoidan (FV) on gene expression in zebrafish embryos. (**A**) Zebrafish embryos were treated 2-dpf with FV, (**B**) lipopolysaccharide (LPS) as a pro-inflammatory agent and (**C**) FV + LPS for 3 h to analyze the gene expression related to VEGF, inflammation, and oxidative stress in comparison to the respective control (Co) groups. Bioactivity of FV was analyzed from FV + LPS against LPS-treated groups. The colored circles indicate, upregulated genes (red), downregulated genes (green), non-significant changes (gray), and flat/unchanged genes (black). The horizontal gray dotted line represents the significance threshold (*p*-value < 0.05), and the vertical red dotted lines represent the fold-change thresholds (log_2_ fold change = ±1), Co (control), *n* = 3.

Embryos treated with FV alone did not significantly alter gene expression compared to the untreated control group (Figure 5A) suggesting no effect of fucoidan on healthy embryos. When embryos were stimulated with LPS, several pro-inflammatory genes were upregulated, including tumor necrosis factor-beta (TNFβ), IL-8, IL-1β, immunoresponsive gene 1, like (IRG1L) and matrix metalloproteinase-9 (MMP9) with Relative Quantification (RQ) values = 2.973, 5.976, 7.006, 7.170 and 8.768 respectively. We also observed G-protein-coupled receptor 84 (gpr84), RQ = 2.263 and tumor necrosis factor-alpha (TNFα), RQ = 2.517 was upregulated but did not reach significance. We also noticed downregulation of Nitric Oxide Synthase 2A (NOS2A) = 0.258 and non-significant downregulation of Superoxide Dismutase 1 (SOD1) with RQ = 0.284. These findings are consistent with a pro-inflammatory response induced by LPS (Figure 5B). The LPS-induced expression pattern was attenuated when embryos were treated with FV and LPS simultaneously (FV + LPS vs. LPS), no longer displaying pro-inflammatory upregulation but showing a trend toward normalization. The expressed genes are shown in Figure 5. The RQ values and their significance of the target genes are listed in Table A1 and Table A2 in the Appendix A.

**Figure 6:**
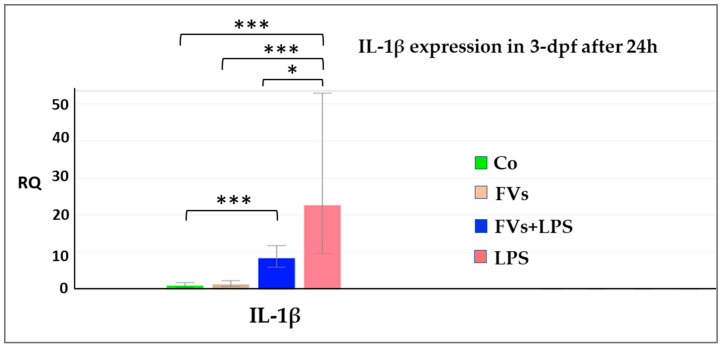
Anti-inflammatory effects of fucoidan (FV) on interleukin-1 beta (IL-1 β) gene downregulation in 3-dpf zebrafish embryos. Zebrafish embryos were treated in vivo with FV, and lipopolysaccharide (LPS) for 24 h. LPS was used as pro-inflammatory agent. The effect of FV was assessed by comparing the FV + LPS group against the LPS-only-treated group. Data represent the influence of FV in LPS-induced inflammatory responses (*p*-value = * 0.05, *** 0.001), Co (control), *n* = 3.

In an additional experiment, 3-dpf embryos were treated with FV and/or LPS for 24 h, followed by single-gene RT-qPCR analysis. Similar to our results above, we found LPS significantly upregulated IL-1β expression, RQ = 22.695 (*p*-value < 0.001), while this effect was significantly downregulated by FV (Figure 6) with RQ = 8.404 and *p*-value = 0.021 (Table 1), supporting a potential protective effect of FV against LPS-induced inflammatory responses.

### 2.4. In Vivo Retinal Vessel Formation

We further tested FV in 3-dpf for retinal angiogenesis. Embryos were treated with 50 µg/mL of FV, 3 µM of SU5416 (semaxanib) and 2.5 mM of cobalt chloride (CoCl_2_). Evaluation of vessel formation was assessed after 24 h by using a laser-scanning microscope (LSM). Quantitative and qualitative analyses included total vessel area, vessel number, sprouting density, and vessel quality, assessed using Fiji ImageJ software (ImageJ bundled with 64-bit Java 8) and manual evaluation of LSM z-stack images.

**Figure 7:**
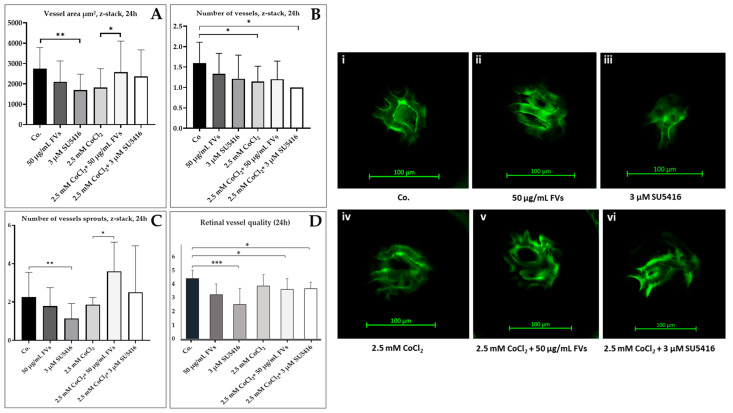
Angiogenic effects of fucoidan (FV) on the vascular system of the zebrafish eye. Three days post-fertilized zebrafish embryos (3-dpf) were treated with FV for 24 h and vascularization in the eyes was analyzed. Quantification includes, (**A**) total vessel area, (**B**) number of vessels, (**C**) number of vessel sprouts/branches, and (**D**) vessel quality. Measurements were performed with ImageJ using LSM z-stack images. Example images are shown from (**i**–**vi**). Scale bar = 100 µm, Significance levels are indicated as *p*-value = * 0.05, ** 0.01, *** 0.001, Control (Co.).

Analysis of total vessel area after ImageJ assessment, revealed SU5416 reduced vessel area significantly from 2861 ± 959 µm^2^ to 1872 ± 628 µm^2^ (*p*-value = 0.004), while CoCl_2_ + FV significantly increased vessel area from 2228 ± 386 µm^2^ to 3297 ± 851 µm^2^ compared with the CoCl_2_-treated group (*p*-value = 0.023, Figure 7A).

A significant reduction in retinal vessel number was observed in embryos treated with CoCl_2_ + SU5416 compared to controls (*p*-value = 0.01). Embryos stimulated with CoCl_2_ alone also showed a significant reduction in vessel number compared to the control group (*p*-value = 0.047). A similar effect was observed in the FV treatments, in which the number of vessels was reduced, but this effect did not reach a significant level (Figure 7B).

We found SU5416 significantly reduced the number of retinal sprouts compared to the control group (*p*-value = 0.009). In contrast, CoCl_2_ + FV treatment significantly increased the number of retinal sprouts against the CoCl_2_-treated group (*p*-value = 0.014) (Figure 7C). Here, FV too showed a reducing effect, but did not reach statistical significance.

We observed a significant deterioration in vessel quality in several treatments. In particular, SU5416 (*p* = 0.001), CoCl_2_ + FV (*p* = 0.040), and CoCl_2_ + SU5416 (*p* = 0.021) showed significant reductions in retinal vessel quality compared to the control groups. However, the groups treated with just FV and CoCl_2_ showed no significant effect on the vascular quality of zebrafish embryos (Figure 7D).

Contrary to our expectations, CoCl_2_ did not induce vessel formation, but rather reduced it compared to the control groups of all treatments. These findings indicate a context-dependent vascular modulatory effect, where FV partially mitigates vessel loss induced by CoCl_2_ rather than acting as a direct anti-angiogenic agent.

### 2.5. In Vivo Antioxidative Stress Effects

**Figure 8:**
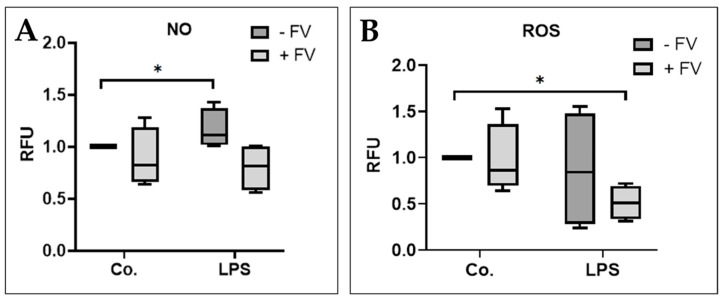
Oxidative stress detection of FV in 3-dpf zebrafish embryos, (**A**) reactive oxygen species (ROS) and (**B**) nitric oxide (NO) production in 3-dpf zebrafish embryos following lipopolysaccharide (LPS) exposure and FV treatment. ROS and NOS levels were assessed using dichlordihydrofluorescein and diaminofluorescein (DCF-DA and DAF-FM). Embryos were treated with 10 µg LPS or in combination with 50 µg/mL FV and compared with control (Co). For non-parametric data, statistical significance was determined using the Mann–Whitney test. Significant values (*p*-value = * 0.05, Co (control), *n* = 4.

To assess the antioxidative stress potential of FV in vivo, nitric oxide (NO) and reactive oxygen species (ROS) production were measured in vivo zebrafish embryos. Three-days-post fertilization (3-dpf) embryos were treated with LPS (10 µg/mL) and FV (50 µg/mL) for 24 h and were compared with the control group. NO and ROS levels were quantified using dichlorofluorescein-based fluorescence assays (Figure 8A).

Our results show LPS treatment induced a significant increase in NO production when compared to the untreated control group (*p*-value = 0.030). This increase was significantly reduced in FV-treated embryos. Similarly, ROS production was elevated following LPS-stimulation and the level was reduced significantly by FV treatment when compared to the untreated control group (*p*-value = 0.030), indicating a reduction in oxidative and nitrosative stress markers in vivo (Figure 8B).

## 3. Discussion

In this study, we evaluated a commercial fucoidan from bladderwrack (*Fucus vesiculosus*) for its bioactivity in both in vitro and in vivo models with respect to its potential application in AMD. In contrast to our previous in vitro studies, this work integrates an in vivo zebrafish model to evaluate pathway-level effects across biological systems. As reported in several studies, the biological activities of fucoidans are greatly influenced by their molecular characteristics such as molecular weight, degree of sulfation, and monosaccharide composition [12,23].

Excessive secretion of VEGF from RPE cells, which may be initiated by oxidative stress, leading to abnormal angiogenesis, is a key driver of pathological angiogenesis and a major contributor to the development of AMD [24,25].

Fucoidans of many kinds from various sources have been extensively studied for VEGF-modulating properties in primary RPE cell models, suggesting their potential relevance for AMD and diabetic retinopathy [12].

Based on the several previous findings by Dörschmann et al. [12] demonstrating efficacy of fucoidans in defined concentration ranges, a concentration of 50 µg/mL was selected in this study as an optimal mid-range dose. Under these conditions, FV significantly reduced VEGF-A level in the RPE cell supernatant, confirming initial observations. As intracellular VEGF-A levels remained unchanged, the observed reduction in extracellular VEGF-A may reflect altered secretion, stability, or trafficking rather than decreased expression. The underlying mechanism was not investigated in this study. Therefore, the observed extracellular reduction in VEGF-A should not be interpreted as a direct decrease in intracellular VEGF-A expression. Instead, FV may influence VEGF-A secretion, release, or extracellular availability.

Additionally, FVs also reduced FST levels in both the supernatant and lysate. The functional relevance of reduced follistatin levels in AMD-related angiogenesis remains unclear and should be considered exploratory. Furthermore, the level of IGFBP-1 that inhibits IGF-stimulated cellular effects such as proliferation and growth signaling [26] was also decreased by FV; however, this reduction was not statistically significant.

We observed an increase in VEGFR-1/FLT-1 and VEGFR-2/KDR levels in the cell lysates compared to the supernatant. These results are related to the biological function of VEGF receptors, which are transmembrane proteins that act within or on the membrane of endothelial cells and are generally not secreted proteins. The increasing content of VEGFR-1/FLT-1 and VEGFR-2/KDR in the lysates indicates changes in receptor synthesis, localization, and signaling activation on the cell surface. Therefore, VEGFR-1/FLT-1 and VEGFR-2/KDR are more abundant in the lysate than in the culture medium [27,28,29,30,31]. Previous studies reported fucoidan to directly counteract VEGF binding to its receptors and inhibit angiogenesis signaling as well as reducing VEGF expression [32]. However, receptor activation and downstream signaling pathways were not assessed in the current study. The VEGFR-1/FLT-1 and VEGFR-2/KDR protein expression was not affected by FV in the lysates. We did not test for receptor activation or inhibition. The differing effects observed for these receptors may be due to differences in molecular weight, as it is known to play a critical role in VEGF modulation and angiogenic activity [12,33].

The use of CoCl_2_ was done previously to enhance angiogenesis and neovascularization [34,35], whereas SU5416 has been used as an anti-angiogenic agent [34]. Contrary to expectations, CoCl_2_ did not enhance angiogenesis but rather led to a reduction in vessel formation. This may be because CoCl_2_ may not exclusively mimic hypoxia-induced angiogenesis. Several studies have shown that, depending on the dose and duration of exposure, CoCl_2_ can induce off-target effects through oxidative stress, such as cytotoxicity, apoptosis, and changes in cell metabolism that are not related to hypoxia signaling [36]. Higher doses of CoCl_2_ disrupt cellular processes leading to changes in gene expression and cell viability that are not observed in hypoxic environments [37]. In contrast to previous reports on fucoidan reducing angiogenesis in vivo models [35,38], in this study, we observed an average reduction in total vessel area and vessel number in embryos treated with FV alone; however, this effect was not significant and weaker than if a commercial anti-angiogenic agent (SU5416) was used. Interestingly, treatment with FV led to a significant restoration of vessel formation, which was reduced by CoCl_2_ exposure, suggesting that FV modulates angiogenic processes under these conditions. However, the vessel quality was not recovered by FV when compared with the CoCl_2_-treated group. This could be due to the toxic effects of CoCl_2_ [39], which could not be reversed by FV. These results demonstrate that FV can modulate vascular responses in a context-dependent manner without affecting healthy embryos, suggesting that FV influences pathological, but not physiological, vessel formation. These findings indicate a context-dependent vascular modulatory effect, where FV partially mitigates vessel loss induced by CoCl_2_ instead of acting as a direct anti-angiogenic agent. While CoCl_2_ may be useful for initial investigations of HIF pathway activation, its toxic effects do not make it a selective hypoxia inducer [39].

Chronic inflammation is a main pathomechanism in both dry and wet AMD, contributing to drusen formation, retinal damage, and disease progression [6]. RPE cells secrete pro-inflammatory cytokines when stimulated with different pro-inflammatory insults [23].

FV significantly inhibited IL-8 secretion levels in response to LPS and PIC treatments, and reduced IL-6 levels against PIC treatments. These stimulus-dependent anti-inflammatory effects are consistent with previous reports [23] and suggest involvement of the Toll-like receptor 3 (TLR3) pathway as RPE cells express TLR-3 which is involved in reducing RPE function [40]. The exact interaction of fucoidan and the TLR-3 is not yet known; however, it could be argued that reduction in RPE65 and phagocytosis could result in sub-lethal injury of the cell, where it would become no longer functional as mentioned by Spaide and Vavvas [41]. The observed cytokine profile therefore suggests an immunomodulatory activity rather than cytokine suppression.

In contrast to its effects on secreted cytokines, FV did not significantly reduce pro-inflammatory cytokines in cell lysates, but a slight reduction in interferon-γ (IFNγ) that was observed on average in all treated groups, suggesting a slight inhibitory effect of FV. The inflammatory response analysis revealed a selective and stimulus-dependent immunomodulatory effect of FV. Significant reduction was primarily observed for IL-8 following LPS and poly(I:C) stimulation, whereas other cytokines were largely unaffected. Moreover, differences between supernatant and lysate data suggest that FV may preferentially modulate cytokine secretion rather than intracellular production. These findings indicate that fucoidan influences specific inflammatory pathways depending on the stimulus context.

Fucoidan has been reported to have anti-inflammatory properties in vitro, and this has also been shown in in vivo studies on zebrafish [42,43,44]. Although most studies did not specifically investigate fucoidan from *F. vesiculosus*. In the present work treatment of 2-dpf zebrafish embryos with 50 µg/mL FV from *F. vesiculosus* fucoidan modulated the expression pattern of several LPS-responsive genes, including IL-8, IL-1β, TNFβ, IRG1L, and MMP9. FV shifted the expression of several LPS-induced genes toward baseline levels without affecting baseline expression in healthy embryos. This indicates that fucoidan affects pathological, but not physiological gene expression. Given the limited sample size, these findings are exploratory and require further validation. The IL-1β expression experiment thus served as a confirmatory study that supported the results of the exploratory screening.

Indeed, Ikeda-Ohtsubo et al. [42] revealed immunomodulatory effects using 500 µg/mL fucoidan from *Cladosiphon okamuranus* over a three-day treatment period. The study demonstrated both pro- and anti-inflammatory gene regulation, supporting the concept of fucoidan as an immunomodulator rather than an anti-inflammatory agent. Similar results were observed with regard to FV, as it increased IL-12 secretion in the supernatant of RPE cells following LPS treatment and decreased it following PIC treatment. In our study, we observed the downregulation of IL-1β in embryos treated with FV at 2-dpf and 3-dpf, suggesting its anti-inflammatory effect. These outcomes demonstrate a clear in vivo anti-inflammatory effect.

ROS and NO from exogenous and endogenous sources play essential roles in physiological signaling; however, excessive production can induce cellular and DNA damage, in a variety of cell types [45], particularly in neuronal tissues [46]. The most common ocular diseases including AMD are mediated by excessive ROS production [47,48].

Fucoidan is widely known for its antioxidant properties [49]. In the present study, *F. vesiculosus* fucoidan (FV) exhibited antioxidant activity in vivo in zebrafish embryos, as evidenced by a reduction in the production of ROS and NO. These results are comparable with previous in vivo zebrafish studies that have demonstrated the antioxidant effects of fucoidan from other brown algae, including *Ecklonia cava* [50] and *Sargassum fusiforme* [43]. These results support the assumption that fucoidans from different algal sources can reduce oxidative stress in vivo, with the extent of this effect possibly depending on structural characteristics such as molecular weight and sulfation patterns [12]. Similar protective effects have been observed in ocular cell lines, including ARPE-19 cells [51].

Overall, this study demonstrates that fucoidan from *F. vesiculosus* exhibits biologically relevant, anti-inflammatory, and antioxidant activities as well as vascular-modulating effects in gene expression and cytokine secretion, in both in vitro and in vivo models relevant to AMD pathophysiology. These data support the concept of FV being a multifunctional, bioactive compound. Compared to mammalian systems, the zebrafish model has a strong capacity for retinal regeneration. Such regenerative capabilities also provide distinctive opportunity to investigate cellular and molecular mechanisms of retina damage and repair, as well as identifying novel therapeutic targets. Nevertheless, embryonic zebrafish models cannot fully reproduce chronic AMD; however, zebrafish embryos are widely used as translational in vivo screening systems for investigating inflammatory and oxidative stress-related mechanisms and for the early evaluation of therapeutic candidates [52,53].

However, several limitations should be acknowledged: 1, The fucoidan used in this study was commercially sourced and not structurally characterized within the present work, although previous characterization data for this preparation are available. 2, Only a single concentration was identified and used as the optimized concentration, limiting dose–response interpretation. Additionally, positive controls were not included in all experimental settings. 3, Zebrafish embryos do not fully reflect the complex and chronic nature of AMD; however, they remain a suitable model for preliminary in vivo screening. 4, Gene expression analysis was conducted with a limited sample size, primarily due to cost constraints and adherence to the principles of the 3Rs (Replacement, Reduction, and Refinement) in animal research. 5, This study did not explore the underlying molecular mechanisms, such as pathways involving VEGF receptor activation, which are critical for understanding the vascular modulatory effects observed.

Future studies should focus on fucoidans with defined molecular weights and known chemical compositions, and optimizing dosing strategies in vivo models to fully elucidate their therapeutic potential for AMD.

## 4. Materials and Methods

### 4.1. In Vitro Experiments

Primary porcine RPE cells were isolated and cultured under sterile laboratory conditions as described by Klettner and Roider [54,55]. The pig eyes were obtained from local slaughterhouses with approval from the animal welfare officer at the University of Kiel. As animals were not sacrificed for research purposes, this procedure complies with the 3R (Replacement, Reduction, Refinement) principles in accordance with the German animal welfare act (TierSchG).

Porcine eyes were processed approximately 4–6 h post mortem. After cleaning and disinfection, eyes were dissected to remove vitreous bodies and retina. RPE cells were detached by enzymatic digestion using trypsin (PAN-Biotech GmbH; #P10-021100) and trypsin/EDTA (PAN-Biotech; #P10-020100). Cells were collected, washed and cultured in HyClone DMEM medium supplemented with 10% fetal bovine serum, 1% penicillin/streptomycin, 5% sodium pyruvate and 1% non-essential amino acids. Unless otherwise stated, all cell culture reagents were obtained from PAN-Biotech GmbH (Aidenbach, Germany).

Cells were seeded at a density of 1.0 × 10^6^ cells/mL into 12-well plates (1mL/well) or 24-well plates (500 µL/well; Sarstedt AG & Co. KG, Nümbrecht, Germany), #83.3922.005). The cultures were fed regularly and maintained at 37 °C in a humidified atmosphere with 5% CO_2_. Experiments were performed after at least two weeks, when cells reached confluence and exhibited characteristic RPE morphology.

Fucoidan (FV) derived from *F. vesiculosus* was obtained from Sigma Aldrich, with a reported purity of ≥95%, and was dissolved in water. FV used in this study had previously been chemically characterized by Dörschmann et al. [51]. The analysis results indicated a high fucose content of 86.2 mol% and a significant degree of sulfation (0.61, arbitrary units), with no protein contamination and a molecular weight of approximately 52 kDa, according to the supplier’s specifications.

Confluent primary RPE cells were first pre-treated with 50 µg/mL FV for 30 min followed by 10 µg/mL of LPS (Merck, Darmstadt, Germany; #L4524), 10 μg/mL polyinosinic:polycytidylic acid (poly(I:C), Tocris Bioscience, Bristol, UK; #4287/10) or 25 µM of erastin, (Cayman Chemical, Ann Arbor, MI, USA; #CAY17754) for 3 days. After incubation the cell culture supernatants were collected, and cells were lysed for Multiplex Luminex Assays. LPS and poly(I:C) were used as pro-inflammatory agents (TLR4 and TLR3 agonists, respectively), while erastin was included as a ferroptosis-inducing agent to simulate oxidative stress-related cell damage. Only one concentration of FV with the combination of other stressors was tested after optimization.

Inflammatory and angiogenesis biomarker concentrations in RPE cell supernatants and lysates were quantified using ProcartaPlex™ Multiplex Immunoassays for inflammation and angiogenesis (Thermo Fisher Scientific, Waltham, MA, USA) according to the manufacturer’s instructions. Measurements were performed using a Luminex^®^ 200 Research Use Only (RUO) System equipped with w/xPONENT^®^ software version 4.3 (LX200-XPON-RUO).

### 4.2. In Vivo Experiments (Zebrafish Model)

The wild-type zebrafish (*Danio rerio*) of the AB/TÜ strain were used for gene expression analyses. Fish were maintained under standard laboratory conditions. Adult zebrafish of age 4 to 12 months of random sex were used for all experiments. Fertilized eggs were collected and incubated for 48 h (2-dpf).

For each experimental condition about 10 to 11 of 2-dpf embryos were treated with FV (50 µg/mL) and/or LPS (30 µg/mL) in 12-well plates for 3 h. Additional experiments were also performed for the expression of the target gene, using 2-dpf embryos treated for 24 h. Embryos were randomly assigned to treatment groups. Each treatment included a control group and was repeated at least three times. After the treatment, embryos were washed and homogenized in 300 µL TRIzol™ reagent (Thermo Fisher Scientific) for RNA extraction. For RNA isolation, in brief, 300 µL of chloroform was added to the TRIzol sample, mixed and centrifuged at 12,000× gravity (*g*) at 4 °C for 15 min to separate the phases. The transparent phase was transferred to a new eppendorf tube. Then the RNA was precipitated by adding 300 µL of isopropanol, incubated for 10 min at room temperature and centrifuged at 12,000× *g* for 10 min at 4 °C. The white RNA pellet at the bottom of the tube was washed three times with 70% ethanol, briefly air-dried, and dissolved in 20 µL RNase-free water.

Transgenic Tg(fli1:EGFP)y1 (AB) 2-dpf zebrafish embryos were used to assess retinal vessel formation. Ten to eleven 2-dpf embryos per well in a 12-well plate were treated with 50 µg/mL of FV, 3 µM of SU5416 (semaxanib), or 2.5 mM of cobalt chloride (CoCl_2_), or left untreated for 24 and 48 h as a control group.

For LSM imaging, 10 embryos per condition were stimulated for 24 and 48 h. Three to five randomly selected embryos per condition were embedded in ultra-pure low-melting-point agarose in a glass-bottom Petri dish and oriented with the eye facing upward. The GFP stained retinal vessels were visualized using ZEISS LSM 880 laser scanning confocal microscope (Carl Zeiss Microscopy GmbH, Jena, Germany) controlled by ZEN 2.3 SP1 software. Quantitative analysis of retinal vessel area was performed using Fiji ImageJ software on z-stack images from LSM. Vessel number, branching (sprouting), and assessment of vessel quality were performed manually. Vessel quality was assessed visually using a scale from 0 to 5, where 0 indicates severely damaged vessels and 5 indicates healthy, intact vessels. The evaluation of data was performed using Fiji ImageJ and MS Excel. Image analysis was performed in a blinded manner.

For oxidative stress analysis we used fluorescence-based assays as indicators of reduced oxidative and nitrosative stress. For nitric oxide (NO) detection 10 to 15 3-dpf wild type (AB) embryos were treated with FV (50 µg/mL) for 30 min followed by LPS (10 µg/mL), for 24 h in a 12-well plate. At least 5 embryos per condition were transferred to flat-bottom 96-well plates (one embryo per well), stained with DAF-FM dye for 30 min at 28 °C, washed and anesthetized with low-dose tricaine and analyzed using a TECAN Spark® multimode microplate reader (Tecan Trading AG, Männedorf, Switzerland) with SparkControl™ software version 3.1.

For reactive oxygen species ROS detection, 3-dpf embryos were pre-treated with FV (50 µg/mL) for 30 min followed by LPS (10 µg/mL) or H_2_O_2_ (150 µM) for 24 h in a 12-well plate. Embryos were stained with DCF-DA for 1 h in the dark at 28 °C, transferred to round-bottom 96-well plates (one embryo per well), washed, anesthetized in tricaine, and the fluorescence was measured using the TECAN Spark microplate reader. At least three experiments were performed per condition.

### 4.3. Real-Time Polymerase Chain Reaction

The purity and concentration of RNA was assessed by using NanoDrop™ One (Thermo Fisher Scientific). RNA was transcribed into cDNA by using the High-Capacity cDNA Reverse Transcription Kit (Thermo Fisher Scientific; #4368814).

Real-time PCR (QuantStudio3) was performed with TaqMan™ Fast Advanced Master Mix (Thermo Fisher Scientific; #4444557) as described in the instructions of the Master Mix. Single-gene assays and a customized zebrafish gene expression array (96 genes) were purchased from ThermoFisher and used for multiple gene expression analysis. The gene array contains 96 genes including housekeeping endogenous controls such as actin beta 1 (ACTB1), beta-2-microglobulin (B2M), glyceraldehyde-3-phosphate dehydrogenase (GAPDH), Hypoxanthine-guanine phosphoribosyltransferase 1 (HPRT1), Ribosomal protein large, P0 (RPLP0), TATA-box binding protein (TBP), and genes associated with inflammatory signaling, oxidative stress responses and VEGF-mediated pathways (Appendix A, Table A1 and Table A2). The 96-gene expression panel was intended as an exploratory screening approach, no correction for multiple testing was applied. Relative gene expression was calculated using the ΔΔCT method [56]. Reference samples were LPS and control, set to RQ = 1.0, respectively. The screening analysis was therefore interpreted as exploratory and hypothesis-generating rather than a confirmatory single-gene differential expression analysis.

### 4.4. Statistical Analysis

Data were evaluated and expressed as mean ± standard deviation (SD) for parametric data as well as median and interquartile range for non-parametric data. Biological replicates represent independent experiments using separate cell isolations or embryo batches. Sample size varied depending on experimental design constraints. Microsoft Excel from Microsoft Office 2010 (Redmond, WA, USA) was used for basic data processing and graph generation. GraphPad Software, Inc., San Diego, CA, USA, (version 9.1.1) was used for non-parametric data and more advanced statistical analyses. Normality of data distribution was assessed by using the Shapiro–Wilk test. The parametric data were analyzed by using analysis of variance (ANOVA) followed by Student’s *t*-test or one-sample *t*-tests, while the non-parametric data were evaluated by using Kruskal–Wallis tests followed by Mann–Whitney post hoc tests. RT-qPCR data were analyzed using Thermo Fisher Connect, in which Student’s *t*-test was applied. Bio groups with *p*-value < 0.05 were considered to be statistically significant.

### 4.5. Animal Ethics Statement

The zebrafish used in this study were bred, raised, and kept in accordance with FELASA guidelines and all relevant ethical regulations [57]. All procedures complied with local authorities and German animal welfare legislation and EU Directive 2010/63/EU on the protection of animals used for scientific purposes. The housing and breeding followed the standards of the international “Principles of Laboratory Animal Care” (NIH no. 86-23, revised 1985).

## 5. Conclusions

This study investigates the bioactive properties of commercially available fucoidan from brown algae *Fucus vesiculosus* with regard to pathomechanisms relevant to AMD. The influence of fucoidan on retinal pigment epithelial cells in vitro and on zebrafish in vivo models was investigated. These findings suggest that fucoidan modulates angiogenesis, inflammation, and oxidative stress-associated processes, including the reduction in VEGF-A secretion, the mitigation of selected pro-inflammatory responses, and the decreased production of ROS and NO. In vivo, fucoidan also demonstrated potential anti-inflammatory and antioxidant effects and mitigated CoCl_2_-induced changes in retinal vessel formation. Overall, these results suggest that fucoidan could be a promising multifunctional candidate for further investigation in the context of AMD and the associated pathogenic mechanisms. However, further studies are needed to understand the underlying cellular mechanisms, and to validate these effects.

## Figures and Tables

**Table 1:** Gene expression analysis in 3-dpf zebrafish embryos treated with lipopolysaccharide (LPS) as a pro-inflammatory agent, and with FV + LPS for 24 h. The bioactivity of FV was assessed by comparing the FV + LPS group against the LPS-only-treated group. Data represent the effect of FV in LPS-induced inflammatory responses (*p*-values < 0.05 were considered nominally significant).

Bio Group Name	Target Name	Rq	Rq Min	Rq Max	*p*-Value vs. Co	*p*-Value vs. LPS
Control	IL-1β	1.0	0.548	1.825	1.000	0.000
LPS	IL-1β	22.695	9.73	52.932	0.000	1.000
FV	IL-1β	1.348	0.778	2.336	0.288	0.000
LPS + FV	IL-1β	8.404	5.975	11.822	0.000	0.021

## Data Availability

Data are available on request.

## Appendix A

### In Vivo Gene Expression Data for Section 2.3 and Section 4.3

In this section, supporting gene expression of 2-dpf zebrafish embryos treated with LPS as a pro-inflammatory agent and FV for 3 h. The bioactivity of FV was assessed by comparing the FV + LPS group against the LPS-only-treated group. Data represent the effect of FV in LPS-induced inflammatory responses (Table A1 and Table A2).

**Table A1:** List of all target genes and gene expression data of 2-dpf embryos treated with LPS vs. control group. LPS = lipopolysaccharide (LPS), Co = control, Rq = Relative Quantification, Rq Min = Relative Quantification Minimum, Rq Max = Relative Quantification Maximum. A *p*-value < 0.05 is considered statistically significant.

Biological Group	Target Genes	Rq	Rq Min	Rq Max	*p*-Value vs. Co
LPS	RNA control	-	-	-	NaN
LPS	actb1	-	-	-	NaN
LPS	angpt2b	1.071	1.011	1.134	0.691
LPS	atoh7	1.110	0.976	1.262	0.467
LPS	b2m	1.083	0.866	1.355	0.850
LPS	c9	1.041	0.864	1.255	0.886
LPS	cat	1.158	1.015	1.320	0.086
LPS	cetp	1.076	0.847	1.368	0.569
LPS	cfb	1.237	0.904	1.693	0.724
LPS	cfhl2	1.161	0.737	1.829	0.609
LPS	cldn3c	1.172	1.027	1.337	0.379
LPS	clu	1.141	0.943	1.380	0.599
LPS	cp	0.934	0.652	1.340	0.779
LPS	cryaa	1.033	0.923	1.156	0.519
LPS	csf1a	1.080	0.948	1.230	0.450
LPS	csf1b	0.915	0.693	1.209	0.447
LPS	ctsd	1.073	1.000	1.151	0.549
LPS	cxcl8 (il8)	5.976	1.978	18.053	0.014
LPS	fancg	1.219	1.021	1.455	0.089
LPS	faslg	0.601	0.235	1.538	0.603
LPS	fbln5	1.097	0.988	1.219	0.097
LPS	flt1	1.187	1.038	1.357	0.095
LPS	flt4	1.059	0.919	1.221	0.562
LPS	gapdh	-	-	-	NaN
LPS	gfap	1.084	1.020	1.152	0.086
LPS	gpr84	2.263	0.113	45.277	0.643
LPS	gss	1.032	0.814	1.309	0.777
LPS	gstp1	1.060	0.919	1.222	0.373
LPS	hif1ab	1.131	1.035	1.236	0.363
LPS	hmox1a	1.054	0.754	1.474	0.843
LPS	hprt1	1.281	1.148	1.430	0.029
LPS	ifng1r (INF-g)	0.871	0.640	1.185	0.413
LPS	igf1	0.964	0.702	1.323	0.795
LPS	il4	1.488	0.929	2.383	0.177
LPS	il6	2.286	0.747	6.996	0.395
LPS	il10	1.706	0.887	3.280	0.075
LPS	il13	0.749	0.521	1.077	0.130
LPS	il21	0.986	0.700	1.391	0.926
LPS	il22	1.157	0.653	2.049	0.620
LPS	il34	1.291	1.192	1.399	0.173
LPS	il11a	0.863	0.495	1.504	0.583
LPS	il11b	0.961	0.748	1.235	0.709
LPS	il1b	7.006	2.895	16.957	0.022
LPS	il6r	1.195	0.929	1.537	0.288
LPS	il6st	1.463	0.879	2.433	0.159
LPS	irg1l	7.170	3.471	14.810	0.002
LPS	kdr	1.307	1.176	1.453	0.194
LPS	lpl	1.108	1.006	1.220	0.102
LPS	mapk10	1.179	1.003	1.386	0.104
LPS	mapk14a (p38)	1.049	0.838	1.312	0.649
LPS	mapk3 (ERK1/2)	1.026	0.882	1.194	0.818
LPS	mapk8b (jkamp, JNK1)	1.006	0.903	1.120	0.958
LPS	mmp2	1.043	0.841	1.295	0.747
LPS	mmp9	8.768	2.553	30.113	0.028
LPS	mpx	0.801	0.565	1.136	0.161
LPS	myd88	1.424	0.895	2.268	0.095
LPS	neurod1	1.088	0.930	1.271	0.271
LPS	nfe2l2a (NRF2)	0.915	0.705	1.188	0.481
LPS	nfkbiaa	2.055	1.733	2.436	0.075
LPS	nfkbiab	1.359	1.017	1.816	0.048
LPS	nkap	1.360	1.214	1.523	0.003
LPS	nos1	1.445	0.984	2.123	0.048
LPS	nos2a	0.258	0.149	0.446	0.010
LPS	nos2b	1.306	0.949	1.798	0.326
LPS	oclna	0.836	0.593	1.180	0.341
LPS	oclnb	1.931	1.639	2.276	0.000
LPS	p65	0.879	0.684	1.130	0.321
LPS	pe6b	0.943	0.787	1.130	0.581
LPS	plg	1.159	1.030	1.305	0.194
LPS	ptgs1	1.144	1.032	1.269	0.329
LPS	ptgs2a	1.350	0.805	2.266	0.215
LPS	rho	1.494	1.013	2.205	0.107
LPS	rpe65a	1.258	1.038	1.525	0.170
LPS	rplp0	-	-	-	NaN
LPS	scarb1	1.057	0.912	1.226	0.621
LPS	serpine1	1.835	0.810	4.153	0.146
LPS	sirt1	1.049	0.859	1.280	0.610
LPS	sod1	0.284	0.006	13.019	0.419
LPS	sod2	1.093	1.004	1.190	0.044
LPS	sparc	-	-	-	NaN
LPS	tbp	1.204	1.052	1.377	0.078
LPS	tgfb2	1.273	1.224	1.324	0.038
LPS	tgfb3	1.246	1.147	1.354	0.010
LPS	tgfbi	1.149	0.848	1.558	0.325
LPS	tlr2	0.847	0.580	1.238	0.373
LPS	tlr3	0.992	0.726	1.354	0.946
LPS	tlr4ba	0.929	0.736	1.172	0.559
LPS	tlr4bb	1.165	0.324	4.194	0.877
LPS	tnfa	2.517	1.371	4.619	0.234
LPS	tnfb	2.973	2.091	4.225	0.001
LPS	tyr	0.943	0.869	1.023	0.467
LPS	vegfaa	1.276	1.115	1.460	0.084
LPS	vegfab	0.989	0.814	1.202	0.925
LPS	vegfr2 (kdrl)	1.171	1.043	1.314	0.193
LPS	vim	1.220	1.128	1.320	0.278
LPS	vldlr	1.278	0.882	1.853	0.136

**Table A2:** Gene expression data of 2-dpf embryos treated with FVs + LPS vs. LPS groups. FV = fucoidan, LPS = lipopolysaccharide (LPS), Rq = Relative Quantification, Rq Min = Relative Quantification Minimum, Rq Max = Relative Quantification Maximum. A *p*-value < 0.05 is considered statistically significant.

Bio Group Name	Target Name	Rq	Rq Min	Rq Max	*p*-Value vs. LPS
FV + LPS	RNA control	-	-	-	NaN
FV + LPS	actb1	-	-	-	NaN
FV + LPS	angpt2b	1.051	0.815	1.356	0.498
FV + LPS	atoh7	0.963	0.838	1.106	0.535
FV + LPS	b2m	1.258	0.787	2.010	0.163
FV + LPS	c9	1.147	0.843	1.561	0.223
FV + LPS	cat	1.086	1.059	1.113	0.149
FV + LPS	cetp	1.271	1.020	1.585	0.050
FV + LPS	cfb	1.195	0.839	1.702	0.253
FV + LPS	cfhl2	0.820	0.566	1.186	0.331
FV + LPS	cldn3c	0.942	0.892	0.996	0.289
FV + LPS	clu	0.841	0.803	0.879	0.052
FV + LPS	cp	1.332	0.892	1.990	0.121
FV + LPS	cryaa	0.856	0.651	1.125	0.112
FV + LPS	csf1a	0.962	0.727	1.273	0.660
FV + LPS	csf1b	0.959	0.932	0.987	0.705
FV + LPS	ctsd	1.102	0.896	1.354	0.167
FV + LPS	cxcl8 (il8)	3.539	0.493	25.420	0.092
FV + LPS	fancg	0.103	0.000	1034.299	0.400
FV + LPS	faslg	0.467	0.344	0.634	0.077
FV + LPS	fbln5	0.950	0.815	1.108	0.373
FV + LPS	flt1	0.886	0.636	1.234	0.261
FV + LPS	flt4	0.908	0.767	1.075	0.187
FV + LPS	gapdh	-	-	-	NaN
FV + LPS	gfap	1.067	1.028	1.107	0.033
FV + LPS	gpr84	15.814	2.918	85.691	0.054
FV + LPS	gss	0.705	0.500	0.993	0.024
FV + LPS	gstp1	1.067	1.008	1.131	0.279
FV + LPS	hif1ab	1.097	1.058	1.137	0.033
FV + LPS	hmox1a	0.968	0.605	1.550	0.852
FV + LPS	hprt1	0.824	0.617	1.100	0.077
FV + LPS	ifng1r (INF-g)	1.089	0.953	1.245	0.502
FV + LPS	igf1	0.935	0.779	1.121	0.611
FV + LPS	il4	1.485	1.011	2.182	0.083
FV + LPS	il6	1.710	0.970	3.013	0.265
FV + LPS	il10	1.386	1.282	1.499	0.235
FV + LPS	il13	0.835	0.615	1.134	0.277
FV + LPS	il21	1.027	0.501	2.105	0.906
FV + LPS	il22	0.870	0.142	5.333	0.787
FV + LPS	il34	0.949	0.779	1.157	0.400
FV + LPS	il11a	1.340	1.267	1.418	0.213
FV + LPS	il11b	1.063	1.013	1.115	0.547
FV + LPS	il1b	1.303	0.493	3.444	0.531
FV + LPS	il6r	0.934	0.744	1.173	0.547
FV + LPS	il6st	0.934	0.840	1.039	0.736
FV + LPS	irg1l	1.701	1.059	2.733	0.112
FV + LPS	kdr	0.972	0.869	1.088	0.572
FV + LPS	lpl	0.921	0.805	1.054	0.133
FV + LPS	mapk10	1.062	1.039	1.085	0.363
FV + LPS	mapk14a (p38)	1.223	0.862	1.735	0.135
FV + LPS	mapk3 (ERK1/2)	1.138	1.035	1.251	0.071
FV + LPS	mapk8b (jkamp, JNK1)	1.041	1.024	1.058	0.364
FV + LPS	mmp2	1.189	1.032	1.370	0.087
FV + LPS	mmp9	1.745	0.778	3.910	0.302
FV + LPS	mpx	0.823	0.633	1.072	0.220
FV + LPS	myd88	0.973	0.805	1.175	0.883
FV + LPS	neurod1	0.922	0.792	1.075	0.275
FV + LPS	nfe2l2a (NRF2)	0.842	0.484	1.466	0.343
FV + LPS	nfkbiaa	0.975	0.610	1.557	0.851
FV + LPS	nfkbiab	0.955	0.882	1.034	0.693
FV + LPS	nkap	1.108	1.012	1.214	0.065
FV + LPS	nos1	1.029	0.793	1.337	0.858
FV + LPS	nos2a	0.821	0.365	1.849	0.506
FV + LPS	nos2b	1.209	0.984	1.486	0.184
FV + LPS	oclna	1.010	0.910	1.121	0.941
FV + LPS	oclnb	1.215	1.015	1.453	0.032
FV + LPS	p65	0.798	0.384	1.657	0.322
FV + LPS	pe6b	0.914	0.860	0.972	0.242
FV + LPS	plg	1.070	0.934	1.225	0.255
FV + LPS	ptgs1	0.953	0.803	1.131	0.419
FV + LPS	ptgs2a	0.897	0.765	1.050	0.602
FV + LPS	rho	1.085	0.894	1.318	0.610
FV + LPS	rpe65a	0.866	0.735	1.020	0.117
FV + LPS	rplp0	-	-	-	NaN
FV + LPS	scarb1	0.787	0.637	0.971	0.015
FV + LPS	serpine1	0.739	0.696	0.784	0.365
FV + LPS	sirt1	0.935	0.889	0.983	0.412
FV + LPS	sod1	5.577	5.459	5.697	0.279
FV + LPS	sod2	1.058	0.921	1.215	0.265
FV + LPS	sparc	-	-	-	NaN
FV + LPS	tbp	1.074	0.976	1.181	0.237
FV + LPS	tgfb2	1.019	0.983	1.057	0.294
FV + LPS	tgfb3	0.990	0.939	1.044	0.773
FV + LPS	tgfbi	0.867	0.774	0.971	0.267
FV + LPS	tlr2	1.155	1.037	1.286	0.358
FV + LPS	tlr3	0.965	0.675	1.379	0.809
FV + LPS	tlr4ba	1.058	0.942	1.188	0.562
FV + LPS	tlr4bb	0.426	0.064	2.817	0.235
FV + LPS	tnfa	1.763	0.530	5.859	0.179
FV + LPS	tnfb	0.874	0.535	1.427	0.467
FV + LPS	tyr	0.998	0.840	1.186	0.974
FV + LPS	vegfaa	0.954	0.852	1.067	0.432
FV + LPS	vegfab	0.856	0.750	0.976	0.089
FV + LPS	vegfr2 (kdrl)	1.028	0.958	1.103	0.571
FV + LPS	vim	1.031	0.969	1.096	0.386
FV + LPS	vldlr	0.804	0.733	0.881	0.172
